# Anti-DPPX antibody-associated encephalitis with the first detected anti-Yo antibody during one-year follow-up: a case report

**DOI:** 10.3389/fimmu.2026.1792230

**Published:** 2026-09-18

**Authors:** Ling Yang, Lingling Qiu, Min Zhang, Ting Zeng, Chengyin Wang

**Affiliations:** 1 Department of Neurology, The Affiliated Guangzhou Hospital of Traditional Chinese Medicine (TCM) of Guangzhou University of Chinese Medicine, Guangzhou, China; 2 Department of Neurology, The Affiliated Traditional Chinese Medicine Hospital, Guangzhou Medical University, Guangzhou, China

**Keywords:** anti-DPPX antibody, anti-Yo antibody, autoimmune encephalitis, case report, immunotherapy

## Abstract

Anti-dipeptidyl-peptidase-like protein 6 (DPPX) antibody encephalitis is an ultra-rare subtype of autoimmune encephalitis (AE), with no more than 115 cases reported in the literature to date. Anti-Yo antibody-associated encephalitis is also uncommon. No previous case reports have described anti-DPPX antibody-associated encephalitis with anti-Yo antibody first detected during the one-year follow-up. This article presents the first such case, admitted to the Department of Neurology, the Affiliated Traditional Chinese Medicine Hospital, Guangzhou Medical University. The patient presented with acute onset manifested by increased muscle tone, neuronal hyperexcitability, autonomic dysfunction and transient myocardial injury. Anti-DPPX antibodies were detected in both serum and cerebrospinal fluid (CSF) upon initial admission, while anti-Yo antibodies were first detected during one-year follow-up. The patient achieved remarkable clinical remission after receiving immunotherapy with intravenous immunoglobulin (IVIG) combined with corticosteroids. We comprehensively summarize the clinical manifestations, diagnostic workup, therapeutic regimen and long-term follow-up outcomes of this case, and conduct a review of the available literature to investigate the pathophysiological mechanisms underlying these two antibodies. This report aims to raise clinicians’ awareness of rare subtypes of AE, provide effective treatment strategies, and advance the understanding of the heterogeneous spectrum of AE.

## Introduction

Autoimmune encephalitis (AE) is an immune-mediated inflammatory disorder of the central nervous system (CNS) characterized by diverse clinical manifestations and distinct antibody-specific pathogenic mechanisms (1–3). AE-related antibodies are categorized into two groups: cell surface antibodies (e.g., anti-NMDAR, anti-LGI1, anti-DPPX, anti-CASPR2, anti-D2R) and intracellular antibodies (e.g., anti-Yo, anti-Hu, anti-GAD65); the latter are often associated with paraneoplastic etiologies (3).

Anti-DPPX antibody encephalitis was first reported by Boronat et al. in 2013 (4). DPPX serves as a regulatory subunit of the KV4.2 potassium channel complex and is widely expressed in the CNS, gastrointestinal smooth muscle, and myocardium (5). Clinical hallmarks include CNS hyperexcitability (e.g., increased muscle tone, myoclonus), cerebellar ataxia, and systemic symptoms such as diarrhea, constipation, and weight loss (4, 5). Patients with coexisting other neuronal autoantibodies are even rare; only a few case reports have described concurrent positivity with anti-CASPR2 (6) or anti-D2R antibodies (7).

Anti-Yo antibody (also known as anti-Purkinje cell antibody) targets cerebellar degeneration-related protein 2 (CDR2), expressed in cerebellar Purkinje cells, spermatogonia, and certain brainstem regions (8). It binds to intracellular Purkinje cell antigens, triggering an immune cascade reaction that ultimately leads to neuronal loss (9). As a typical paraneoplastic antibody, it is predominantly associated with cerebellar dysfunction (10). Clinical manifestations typically include dizziness, progressive ataxia, dysphagia, dysarthria, and nystagmus; over 90% of cases are accompanied by underlying malignancies, most frequently breast or ovarian carcinoma (11).

To date, anti-DPPX antibody-associated encephalitis with anti-Yo antibody first detected during one-year follow-up has not been previously described. This unique case expands the phenotypic spectrum of AE and raises important questions about the pathophysiological mechanisms of these two antibodies. We present a detailed case analysis focusing on diagnostic challenges, therapeutic effects, and long-term outcomes, supplemented by the literature review to contextualize the clinical and scientific significance.

## Case presentation

### Clinical history

A 57-year-old male presented with acute-onset limb weakness and unsteady gait over 2 days. Associated symptoms included fatigue, limb stiffness, leftward-deviated gait, dysphagia with choking, slurred speech, dizziness, heavy-headedness, right thigh pain, productive cough with white sputum, shortness of breath, occasional chest tightness, and profuse sweating. He denied abdominal pain, diarrhea, or chest pain. Additional complaints included poor appetite, insomnia, constipation, and a 3-3.5 kg weight loss over the past week. He has no significant past medical history.

### Physical examination on admission

Physical examination revealed temperature 36.5°C and blood pressure 120/70 mmHg. Cardiopulmonary and abdominal examinations were unremarkable. Neurological examination revealed dysarthria, difficulty with tongue protrusion and mouth opening, diminished bilateral gag reflex, mildly increased muscle tone in all limbs, and left limb muscle strength graded as 5− (Medical Research Council scale). Pathological reflexes were absent, with no other focal neurological deficits.

### Initial laboratory and imaging findings

Laboratory investigations showed elevated tumor markers: carcinoembryonic antigen (CEA) 28.79 ng/mL (reference range: 0-5 ng/mL) and carbohydrate antigen 19-9 (CA 19-9) 41.81 U/mL (reference range: 0-37 U/mL). CK-MB mass assay was elevated at 6.27 ng/mL (reference range: 0-5 ng/mL). Complete blood count, electrolytes, liver and renal function, lipid profile, other cardiac enzymes, rheumatologic markers, non-neuronal autoantibodies, and humoral immunity panels were within normal limits.

Cranial Computed Tomography (CT) revealed a hypodense area in the left cerebellar hemisphere, suggestive of old encephalomalacia. Chest CT showed no space-occupying lesions, and abdominal CT revealed no significant abnormalities.

### Disease progression and re-evaluation

A preliminary diagnosis of acute cerebral infarction was made, and aspirin (100 mg/day) and other relevant medications were initiated. However, the patient’s condition deteriorated progressively over the subsequent 3 days, with worsening dysphagia, weak cough, severe dyspnea, generalized profuse sweating, marked muscle rigidity, intermittent tonic-clonic-like episodes, migratory pain, lower limb weakness (inability to ambulate), aggravated dysarthria, and palpitations.

Physical examination revealed difficulty opening the mouth and protruding the tongue, severe dysarthria, significantly increased limb muscle tone, rigidity of abdominal and back muscles, lower limb strength graded as 3, and bilateral ankle clonus (**Supplementary Figures 1A, B**). Brain Magnetic Resonance Imaging (MRI) showed no acute infarction and confirmed left cerebellar encephalomalacia near the vermis (**Supplementary Figure 2**). Electroencephalography (EEG) was normal and Electromyography (EMG) showed no abnormal spontaneous activity.

Serial measurements of cardiac biomarkers revealed transient elevation followed by resolution (**Table 1**). Serial 15-lead electrocardiograms and continuous cardiac rhythm monitoring revealed no persistent ischemic changes or sustained malignant arrhythmias. Transthoracic echocardiography demonstrated left ventricular ejection fraction of 67%, without regional wall motion abnormalities or apical ballooning. Cardiology consultation excluded acute coronary syndrome and Takotsubo cardiomyopathy. D-dimer remained within normal range. Blood gas analysis showed no hypoxemia or carbon dioxide retention, and chest imaging detected no consolidation consistent with pneumonia.

**Table 1:** Dynamic changes of cardiac biomarkers during hospitalization.

Time point	CK(50-310 U/L)	CK-MB(0–5 ng/mL)	TnI(0–53.53 pg/mL)	Myoglobin(0–110 ng/mL)	Pro-BNP( 0-125 pg/mL)
Admission day 1	*304.6*	*6.27*	*5.19*	*145.34*	*170.0*
Admission day 3 (peak)	*1822.7*	*14.81*	*335.78*	*610.25*	*4351.0*
Admission day 7	*480.7*	*14.04*	*8.74*	*429.37*	*393.0*
Admission day 14	*188.1*	*8.12*	*8.04*	*156.47*	*134.0*
Admission day 20	*41.0*	*3.53*	*42.94*	*88.46*	*526.0*

Serial testing revealed transient elevation of multiple cardiac biomarkers, peaking on admission day 3, followed by gradual spontaneous decline. In the absence of anti-ischemic therapy, most biomarkers returned to or approached their respective reference ranges; Pro−BNP remained mildly elevated with fluctuations, yet showed a marked decrease from its peak level, indicating reversible myocardial injury.

Cerebrospinal fluid (CSF) (opening pressure 80 mmH_2_O) was clear and colorless. Routine tests including cell count, glucose, protein, and chloride were normal. No cryptococci or acid-fast bacilli were detected; syphilis testing was negative. Serum and CSF autoimmune encephalitis antibody panels were tested using the cell-based indirect immunofluorescence assay (IFA). Results showed strong positivity for anti-DPPX IgG in both serum (titer 1:100) and CSF (titer 1:3.2) (**Figures 1A, B**).

**Figure 1:**
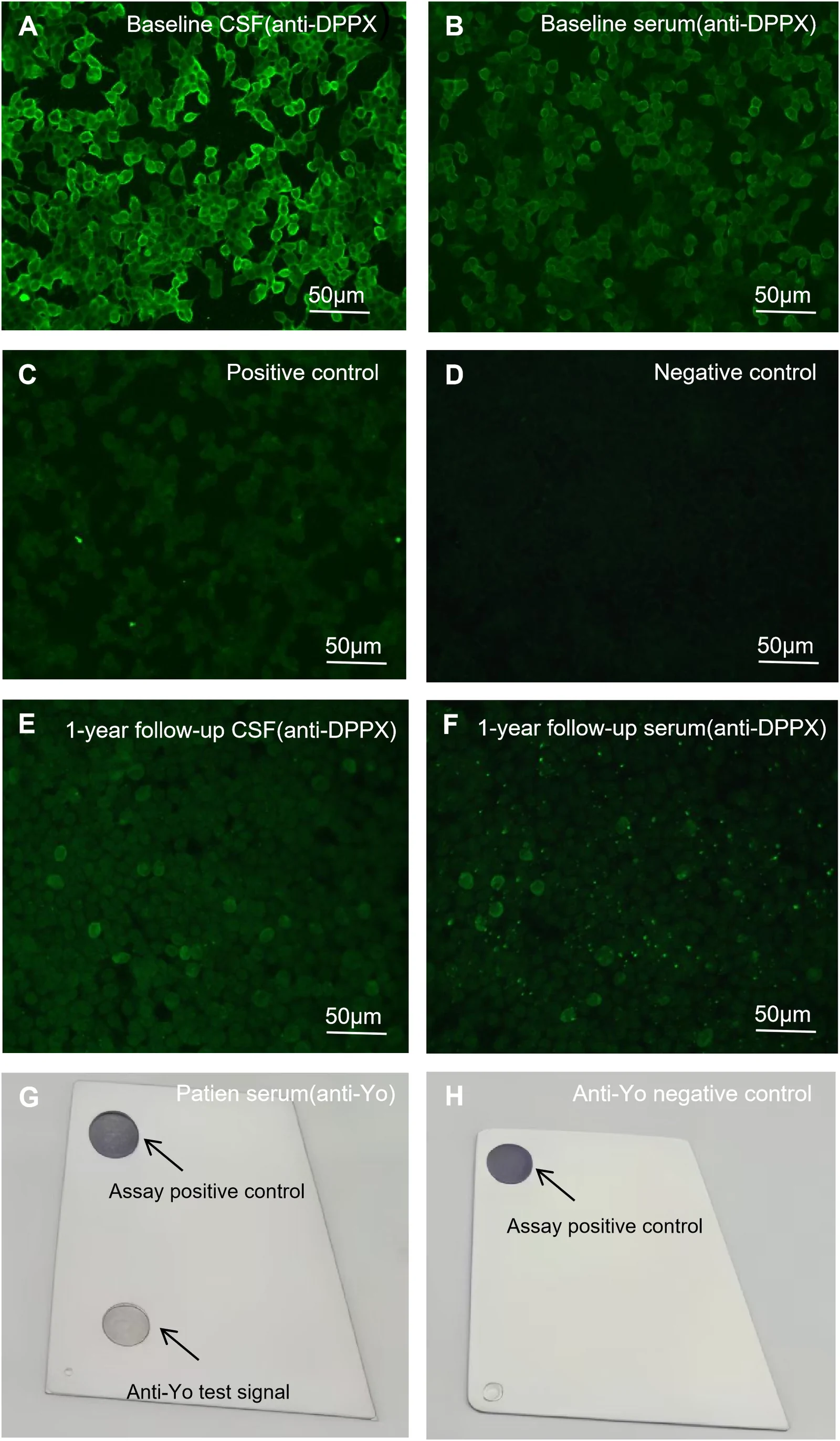
Cell-based assay (CBA) and dot immunobinding assay (DIBA) results of autoantibodies at baseline and the 1-year follow-up. Autoantibody profiles in serum and cerebrospinal fluid at baseline and the 1-year follow-up. At baseline, anti-DPPX antibodies were positive in both serum and cerebrospinal fluid (CSF) using cell-based assay (CBA), with titres of 1:100 in serum and 1:3.2 in CSF. Intracellular paraneoplastic antibodies including anti-Yo were not tested at this time point. At the 1-year follow-up, anti-DPPX antibodies remained positive in CSF [**(E)**, titre 1:1] and serum [**(F)**, titre 1:100] as detected by CBA. For DIBA results, explicit on-image labels mark the assay positive control signal and anti-Yo signal in panel **(G)** (patient’s serum at 1-year follow-up). A distinct positive dot signal was observed at the anti-Yo antigen position in the patient’s serum **(G)**, whereas only the assay positive control signal was visible and no specific anti-Yo signal was detected in the negative-control sample **(H)**. Anti-Yo antibody testing was conducted using the MT152-16 paraneoplastic neurological syndrome autoantibody IgG kit (Maiyuan Biotechnology Co., Ltd., Shanxi, China).

### Treatment and follow-up

Based on serological findings and clinical manifestations, the diagnosis was revised to anti-DPPX antibody encephalitis. The patient was immediately initiated on first-line immunotherapy: intravenous immunoglobulin (IVIG) 0.4g/kg/day for 5 days, followed by methylprednisolone 1000 mg/day for 3 days (pulse therapy), then a gradual tapering regimen (500 mg/day for 3 days, 250 mg/day for 3 days, 120 mg/day for 3 days) and transition to oral prednisone (60 mg/day). Mycophenolate mofetil (0.5 g twice daily) was added as a second-line agent for long-term maintenance.

The patient’s symptoms improved significantly after immunotherapy initiation. After 20 days, he achieved complete clinical recovery, with resolution of muscle rigidity, dyspnea, dysphagia, and weakness (**Supplementary Figures 1C, D**). Most biomarkers returned to or approached reference ranges. Pro-BNP remained mildly elevated with fluctuations, yet showed a marked decrease compared with the peak level (**Table 1**). He was discharged with oral prednisone (60 mg/day, to be tapered gradually) and mycophenolate mofetil (0.5 g twice daily).

During regular follow-up, the patient remained asymptomatic. Mycophenolate mofetil was discontinued 6 months after discharge, and prednisone was tapered to 5 mg/day for long-term maintenance.

One-year follow-up, the patient remained clinically asymptomatic (**Supplementary Figures 1E, F**). All cardiac biomarkers fully recovered to normal reference ranges. Serum anti-DPPX IgG remained positive (titre 1:100) and CSF anti-DPPX IgG positive (titre 1:1) (**Figures 1E, F**), with first detected serum anti-Yo IgG positivity (**Figure 1G**) (dot immunobinding assay (DIBA)). Autoantibody profiles at baseline and 1-year follow-up are summarized in **Table 2**. Chest and abdominal CT showed no evidence of malignancy. The final diagnosis was revised to anti-DPPX antibody-associated encephalitis with the first detected anti-Yo antibody during one-year follow-up. The patient continues oral prednisone 5 mg once daily for maintenance, with no adverse effects reported (**Figure 2**).

**Table 2:** Autoantibody profiles in serum and cerebrospinal fluid at baseline and the 1-year follow-up.

Antibody	Baseline serum	Baseline CSF	Follow-up serum	Follow-up CSF	Assay
Anti-AMPAR1	Negative	Negative	Not tested	Not tested	Cell-based assay
Anti-AMPAR2	Negative	Negative	Not tested	Not tested	Cell-based assay
Anti-AQP4	Negative	Negative	Not tested	Not tested	Cell-based assay
Anti-CASPR2	Negative	Negative	Not tested	Not tested	Cell-based assay
Anti-D2R	Negative	Negative	Not tested	Not tested	Cell-based assay
Anti-DPPX	positive (titre 1:100)	positive (titre 1:3.2)	positive (titre 1:100)	positive (titre 1:1)	Cell-based assay
Anti-GABAARα1	Negative	Negative	Not tested	Not tested	Cell-based assay
Anti-GABAARβ3	Negative	Negative	Not tested	Not tested	Cell-based assay
Anti-GABAAR1	Negative	Negative	Not tested	Not tested	Cell-based assay
Anti-GAD65	Negative	Negative	Not tested	Not tested	Cell-based assay
Anti-GFAP	Negative	Negative	Not tested	Not tested	Cell-based assay
Anti-IgLON5	Negative	Negative	Not tested	Not tested	Cell-based assay
Anti-LG1	Negative	Negative	Not tested	Not tested	Cell-based assay
Anti-mGluR5	Negative	Negative	Not tested	Not tested	Cell-based assay
Anti-MOG	Negative	Negative	Not tested	Not tested	Cell-based assay
Anti-NMDAR	Negative	Negative	Not tested	Not tested	Cell-based assay
Anti-Hu	Not tested	Not tested	Negative	Not tested	Dot immunobinding assay
Anti-Yo	Not tested	Not tested	positive	Not tested	Dot immunobinding assay
Anti-Ri	Not tested	Not tested	Negative	Not tested	Dot immunobinding assay
Anti-Ma2	Not tested	Not tested	Negative	Not tested	Dot immunobinding assay
Anti-CV2	Not tested	Not tested	Negative	Not tested	Dot immunobinding assay
Anti-Amphiphysin	Not tested	Not tested	Negative	Not tested	Dot immunobinding assay
Anti-Ma1	Not tested	Not tested	Negative	Not tested	Dot immunobinding assay
Anti-SOX1	Not tested	Not tested	Negative	Not tested	Dot immunobinding assay
Anti-Tr (DNER)	Not tested	Not tested	Negative	Not tested	Dot immunobinding assay
Anti-Zic4	Not tested	Not tested	Negative	Not tested	Dot immunobinding assay
Anti-PKCγ	Not tested	Not tested	Negative	Not tested	Dot immunobinding assay
Anti-Recoverin	Not tested	Not tested	Negative	Not tested	Dot immunobinding assay
Anti-Titin	Not tested	Not tested	Negative	Not tested	Dot immunobinding assay

Autoantibody profiles in serum and cerebrospinal fluid at baseline and the 1-year follow-up. At baseline, anti-DPPX antibodies were positive in both serum and cerebrospinal fluid (CSF) using cell-based assay (CBA), with titres of 1:100 in serum and 1:3.2 in CSF. Intracellular paraneoplastic antibodies including anti-Yo were not tested at this time point. At the 1-year follow-up, anti-DPPX remained positive in serum (1:100) and CSF (1:1) by CBA. A distinct visible dot signal was observed at the anti-Yo antigen position in serum via Dot immunobinding assay (DIBA) using the MT152-16 paraneoplastic neurological syndrome (PNS) autoantibody IgG kit (Maiyuan Biotechnology Co., Ltd., shanxi, China).

**Figure 2:**
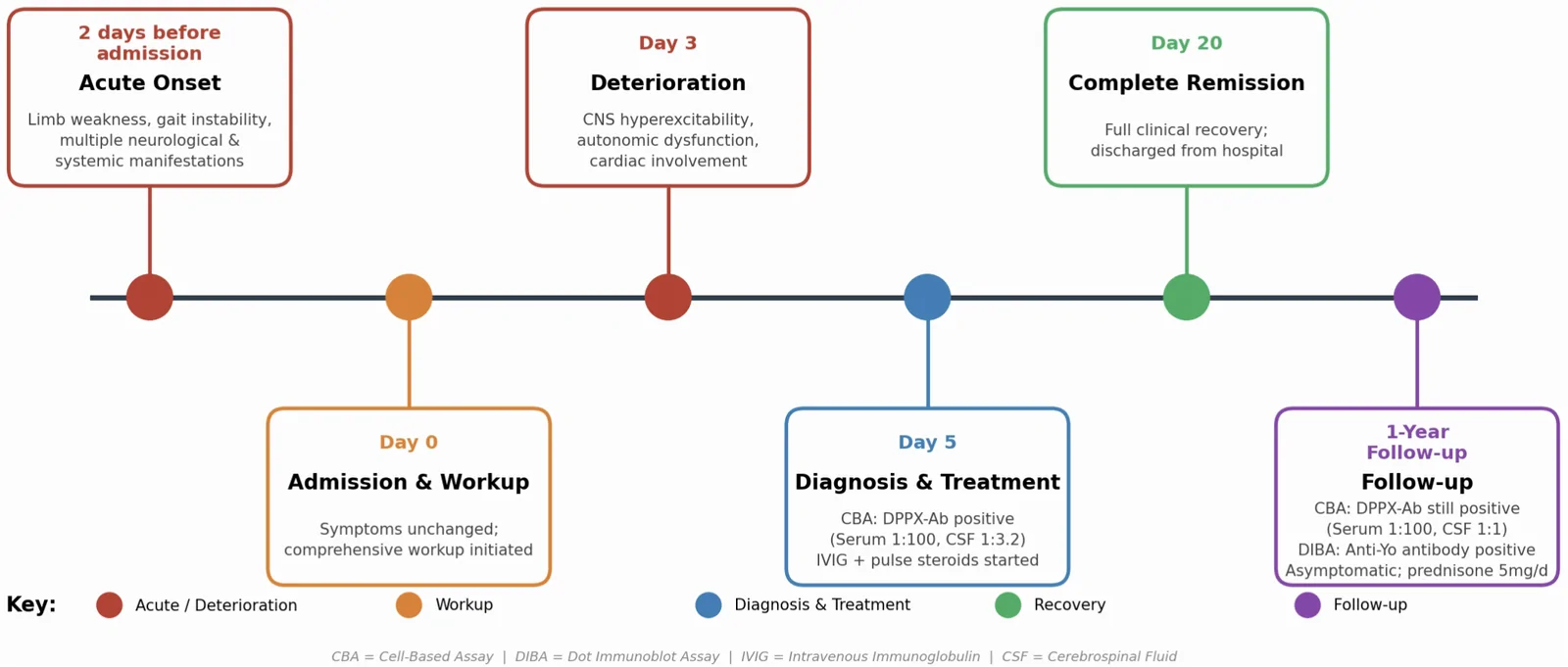
Clinical course and treatment timeline of the patient.

## Discussion

### Novelty and diagnostic challenge

This case report describes the first case of anti-DPPX antibody-associated encephalitis with anti-Yo antibody first detected during the one-year follow-up, expanding the serological heterogeneity of AE. The appearance of these two antibodies in the same patient is particularly notable given their distinct antigenic targets, pathogenic mechanisms, and clinical associations: anti-DPPX antibodies target a cell surface potassium channel subunit, while anti-Yo antibodies target an intracellular antigen (CDR2) and are tightly linked to paraneoplastic syndromes (4, 8, 11).

Initially, the patient presented with gait instability, dysarthria, and vertigo; cranial CT demonstrated chronic cerebellar encephalomalacia, findings that closely mimicked acute cerebrovascular events. Such misdiagnosis remains a frequent pitfall for rare AE subtypes (12). Despite treatment with antiplatelet agents and other cerebrovascular medications, the patient developed progressive clinical worsening. Brain MRI showed no acute lesions, excluding acute stroke. In addition, the patient developed manifestations of central nervous system (CNS) hyperexcitability including muscular rigidity and tonic-clonic seizure-like episodes, accompanied by systemic manifestations such as profuse sweating and palpitations. These clinical deteriorations prompted a comprehensive reassessment of the initial diagnosis and triggered screening for AE-associated autoantibodies. Recent studies highlight the importance of comprehensive autoantibody screening when initial diagnostic considerations cannot fully explain the clinical picture (3). The patient’s clinical features closely align with the classic triad of anti-DPPX antibody-associated encephalitis: gastrointestinal or systemic wasting, cognitive-psychiatric symptoms, and CNS hyperexcitability. Cognitive-psychiatric symptoms are not universal across all cases. Review of the available literature shows normal MRI and CSF findings in most patients with anti-DPPX encephalitis, and the strongest diagnostic evidence relies on DPPX antibody detection in both serum and CSF (5). In this case, confirmed DPPX antibody positivity in both serum and CSF provides highly specific etiological evidence. Anti-DPPX antibody-associated encephalitis is an independent disease diagnosis. By contrast, “anti-DPPX-mediated CNS hyperexcitability syndrome” is not an independent disorder with a well-defined diagnostic framework, and “PERM-like syndrome” only captures partial clinical features. Both lack etiological specificity and diagnostic standardization, and neither can substitute for antibody-based etiological diagnosis. Serum anti-Yo antibody positivity was first detected at the 1-year follow-up. Given the patient’s prominent increased muscle tone, neuronal hyperexcitability and autonomic dysfunction, CNS infection, epilepsy, and stiff-person syndrome were included in the differential diagnosis.Negative CSF microbial assays and systemic infection screening, as well as the lack of infectious lesions on imaging, rendered CNS infection improbable. EEG provided no supportive evidence; combined with clinical features, epileptic disorders were excluded. Despite axial and limb rigidity, anti-GAD65 and other stiff-person spectrum antibodies were negative, and EMG showed no characteristic changes, inconsistent with classic stiff-person syndrome. Other causes of generalized increased muscle tone, neuronal hyperexcitability and autonomic dysfunction were ruled out.

### Pathophysiological significance

The pathophysiological mechanisms of anti-DPPX antibody-associated encephalitis with the first detected anti-Yo antibody during one-year follow-up remain elusive. It is currently thought that the two autoantibodies may share common immune triggers (13) or exert synergistic pathogenic effects (5, 9). The patient’s initial cerebellar symptoms (unsteady gait, dizziness) and imaging-detected cerebellar encephalomalacia foci may be attributable to anti-Yo-mediated Purkinje cell injury. By contrast, acute deterioration, featuring CNS hyperexcitability and cardiac involvement is more readily attributable to anti-DPPX antibody effects. Notably, marked elevation of cardiac biomarkers indicating myocardial injury has not been documented in prior cases of anti-DPPX encephalitis. Markedly elevated cardiac biomarkers were detected during the acute phase. To elucidate the etiology of myocardial injury, common cardiac and systemic triggers were systematically excluded. The patient exhibited no typical angina or persistent ischemic electrocardiographic abnormalities. Troponin and creatine kinase-MB (CK-MB) levels spontaneously normalized in the absence of anti-ischemic therapy, rendering acute coronary syndrome unlikely. A normal D-dimer level and unremarkable pulmonary imaging excluded pulmonary embolism. Although the patient suffered intermittent cough, expectoration and dyspnea, neither pulmonary consolidation nor hypoxemia was identified, and inflammatory biomarkers were only mildly elevated; thus, myocardial injury and respiratory failure attributable to severe infection were ruled out. Transthoracic echocardiography demonstrated preserved left ventricular ejection fraction, normal regional wall motion, and an absence of apical ballooning, thereby excluding stress cardiomyopathy (Takotsubo cardiomyopathy). Continuous rhythm monitoring and 15-lead electrocardiography revealed no sustained arrhythmias or fixed ischemic changes. Cardiology consultation uncovered no evidence of primary cardiac disease. After excluding common causes of myocardial injury, we hypothesize that these cardiac abnormalities stem from neurogenic myocardial dysfunction triggered by binding of anti-DPPX antibodies to myocardial KV4.2 channels, ultimately leading to reversible myocardial injury. One published case described cardiac arrest in a patient with anti-DPPX encephalitis (14), supporting this inference. Nevertheless, this mechanism remains speculative for the present case and cannot be definitively confirmed. Further clinical and experimental investigations are required to validate this hypothesis. Dynamic profiles of cardiac biomarkers are presented in **Table 1**: biomarkers showed transient elevations during hospitalization; most gradually normalized spontaneously or neared reference ranges. Pro-BNP remained mildly elevated with fluctuations but was significantly lower than its peak. The patient was free of associated symptoms (chest tightness, palpitations, chest pain, dyspnea). These findings may be related to residual myocardial burden during recovery from myocardial involvement or stress response fluctuations, with complete normalization at the 1-year follow-up (15).

### Clinical management and prognostic considerations

First-line immunotherapy (high-dose corticosteroids, IVIG, plasma exchange) constitutes the mainstay of AE treatment; second-line agents are recommended for combined therapy in refractory or relapsing cases (16). Our patient achieved full clinical recovery following corticosteroids and IVIG. Existing literature indicates that anti-DPPX encephalitis generally carries a favorable prognosis after immunotherapy, although relapses occur in approximately 23% of patients (17). Some cases are associated with malignancies (18). Chronic cerebellar encephalomalacia on imaging was inconsistent with the typical phenotypic spectrum of anti-DPPX autoimmunity. Given these findings, comprehensive serum antibody screening for paraneoplastic neurological syndromes was conducted at the 1-year follow-up. Anti-Yo antibodies were first detected as positive by DIBA. Anti-Yo antibody-associated encephalitis generally has a poorer prognosis due to its strong association with malignancy and irreversible neuronal loss (11). The favorable outcome may be attributed to early immunotherapy, absence of underlying malignancy, and combined immunotherapy targeting distinct pathogenic pathways.

Despite complete clinical recovery, anti-DPPX antibodies remained persistently positive and anti-Yo antibodies were first detected at the 1-year follow-up. This aligns with prior observations demonstrating that antibody titers do not always correlate with clinical outcomes in AE (19). Accordingly, treatment adjustments should be guided primarily by clinical monitoring rather than antibody titers alone. Although anti-Yo antibody is closely associated with tumors, no malignancy was detected during one year of follow-up. Several potential explanations are proposed herein. First, occult micrometastases or small primary tumors below the detection threshold of conventional imaging cannot be completely excluded. Conventional contrast-enhanced CT lacks sufficient sensitivity to identify tiny early malignancies or microtumors. Second, anti-Yo seropositivity may reflect an atypical non-paraneoplastic immune response unrelated to malignancy. Although anti-Yo antibodies are strongly associated with tumors, rare tumor-negative cases have been documented. Autoantibody production could be triggered by prior infection, inflammatory CNS injury, or systemic immune dysregulation. Under such circumstances, immune activation disrupts self-tolerance and induces anti-Yo antibody generation in the absence of an underlying neoplasm. Third, gender disparities in anti-Yo-associated syndromes merit consideration. Classic anti-Yo paraneoplastic cerebellar degeneration predominantly occurs in female patients with gynecological malignancies. Male anti-Yo-positive individuals constitute a rare subgroup with less consistent association with malignancy. Fourth, the detected anti-Yo antibody reactivity may reflect nonspecific binding or low-affinity antibodies with no definitive pathogenic significance. Serum anti-Yo positivity in this case was identified by dot immunoblot assay (DIBA), without independent confirmation via alternative assays; its clinical significance therefore remains undetermined. Nevertheless, long-term oncological surveillance remains mandatory owing to the robust paraneoplastic propensity of anti-Yo antibodies (11). Based on our team’s long-term clinical experience, an individualized tumor surveillance regimen was devised: annual screening for a minimum of 5 years, comprising contrast-enhanced thoracoabdominal CT and serum tumor marker testing. Prompt whole-body PET-CT is indicated if new-onset neurological symptoms develop or suspicious lesions are detected on routine imaging.

### Literature review and clinical implications

Review of the available literature (PubMed, Embase, Web of Science; search updated to December 2025) using the keywords “anti-DPPX antibody”, “anti-Yo antibody, “ “autoimmune encephalitis, “ and “paraneoplastic neurological syndrome” identified no previously published cases describing anti-DPPX antibody-associated encephalitis with the first detected anti-Yo antibody during one-year follow-up. The rarity of this antibody combination underscores an important clinical reminder: for patients presenting with overlapping cerebellar dysfunction and CNS hyperexcitability, or when initial diagnoses fail to account for disease progression, comprehensive relevant antibody screening covering both cell surface and intracellular neuronal antibodies is essential.

### Limitations

This study has several limitations. First, the patient presented with acute onset characterized by markedly elevated CNS excitability, gastrointestinal autonomic symptoms, and movement disorders, a clinical phenotype highly consistent with anti-DPPX encephalitis. Given this typical presentation, initial screening prioritized neuronal cell-surface autoantibodies using CBA-IFA, while testing for intracellular paraneoplastic antibodies (including anti-Yo antibodies) was not performed at admission. Accordingly, we cannot fully exclude the possibility that anti-Yo antibodies were already present but undetected at disease onset. Pretreatment serum and CSF samples were not preserved, precluding retrospective anti-Yo antibody testing on baseline specimens. Second, antibody testing at different time points relied on distinct laboratory assays. Anti-DPPX antibodies were measured using cell-based indirect immunofluorescence assay (CBA-IFA), whereas follow-up anti-Yo antibody detection was performed via DIBA. In clinical practice, DIBA serves as the conventional first-line assay for screening intracellular paraneoplastic autoantibodies such as anti-Yo. This method exhibits high specificity and favorable reproducibility targeting intracellular antigens including CDR2, with commercially available validated kits for paraneoplastic autoantibody screening (20). Third, anti-Yo antibodies are classic paraneoplastic neuronal antibodies, and serological testing has high diagnostic specificity in typical clinical settings (21). The initial cerebellar symptoms and cerebellar encephalomalacia on imaging in these patients may be attributable to anti-Yo antibody-mediated Purkinje cell damage. However, as serum anti-Yo antibodies were only detected by DIBA, without CSF testing or independent confirmatory assays, their clinical significance remains unclear. Nevertheless, cell-based indirect immunofluorescence and dot immunobinding assay differ in sensitivity and specificity, prohibiting direct comparative analysis of serological results across time points. Nevertheless, CBA-IFA and DIBA differ in sensitivity and specificity, prohibiting direct comparative analysis of serological results across time points. Furthermore, coronary computed tomography angiography (CCTA) was not conducted during hospitalization or long-term follow-up. Although clinical manifestations, electrocardiography, echocardiography, and spontaneous resolution of cardiac biomarkers collectively rendered acute coronary syndrome highly unlikely, occult asymptomatic coronary artery disease cannot be completely ruled out. The absence of CCTA constitutes an important limitation in the cardiac evaluation of this case.

## Conclusion

In summary, we report the first documented case of anti-DPPX antibody-associated encephalitis with anti-Yo antibody first detected during the one-year follow-up, expanding the known phenotypic heterogeneity of this disease. This case illustrates major diagnostic challenges associated with rare AE subtypes, whose clinical manifestations can closely mimic common neurological disorders including cerebrovascular disease. Timely, comprehensive testing for neuronal autoantibodies is therefore critical to avoid misdiagnosis and facilitate recognition of these rare entities. Notably, first-line immunotherapy combining high-dose corticosteroids and IVIG can lead to favorable clinical outcomes even in patients with two distinct autoantibodies, as demonstrated in our patient. Given the strong paraneoplastic association of anti-Yo antibodies, long-term follow-up incorporating serial clinical assessment and rigorous tumor surveillance is imperative for optimal patient management. This report deepens our understanding of the heterogeneous spectrum of AE, provides valuable diagnostic and therapeutic guidance for clinicians, and highlights the need for further research to elucidate the pathophysiological mechanisms driving the generation of multiple neuronal autoantibodies in AE.

## Data Availability

The original contributions presented in the study are included in the article/**Supplementary Material**. Further inquiries can be directed to the corresponding author.
