# Supplementary material for: Anti-DPPX antibody-associated encephalitis with the first detected anti-Yo antibody during one-year follow-up: a case report
Source: Front Immunol. 2026 Sep 18;17:1792230. doi: 10.3389/fimmu.2026.1792230 (PMC13630750; doi:10.3389/fimmu.2026.1792230)
Supplement: Supplementary file 1 [file Image1.pdf]

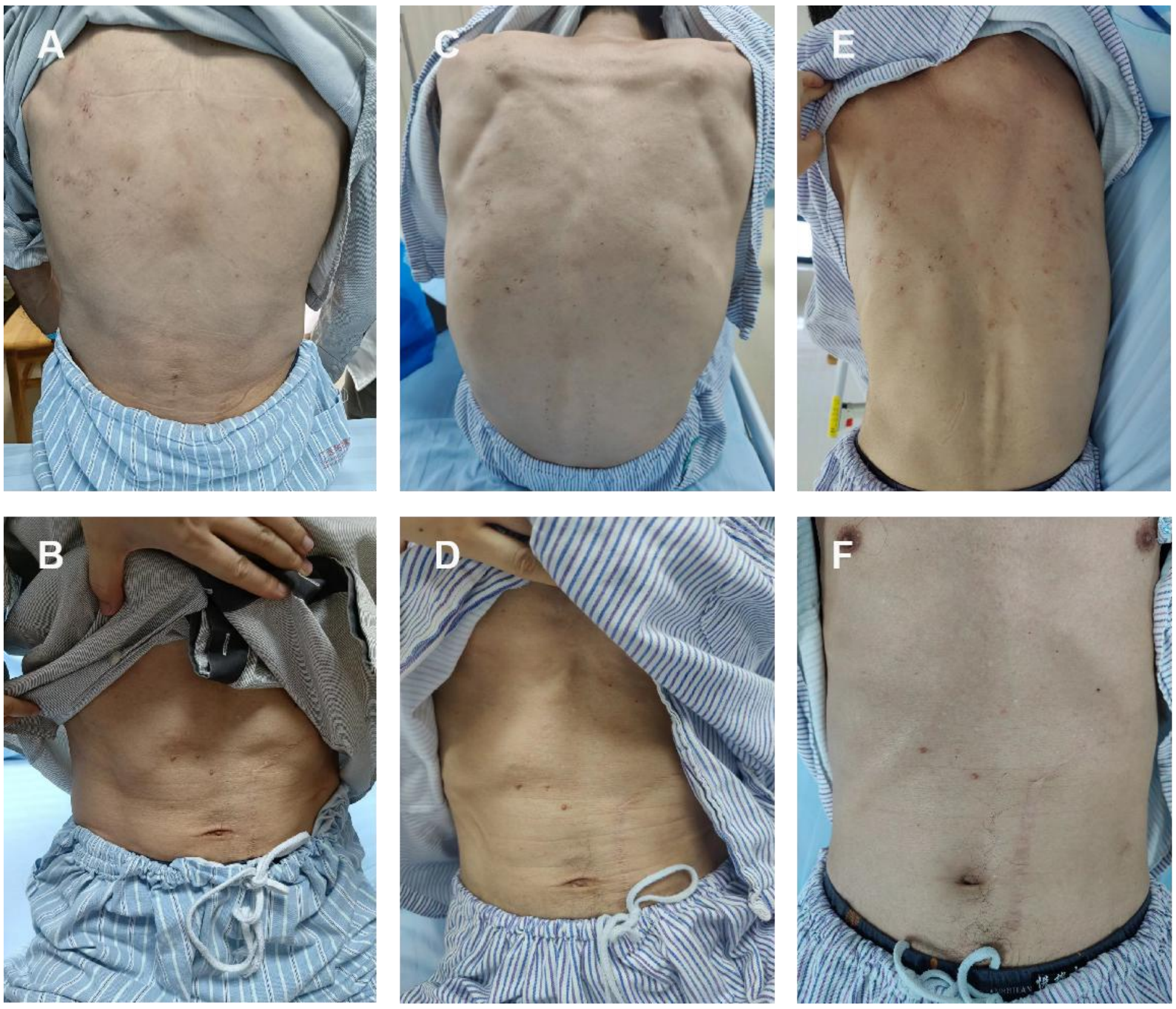

Supplementary Figure 1. Comparison of abdominal and lumbar muscle rigidity before and after treatment. A and B show marked rigidity prior to therapy; C and D demonstrate marked improvement at discharge; E and F confirm complete resolution with no residual stiffness at the 1-year follow-up.
