# Supplementary material for: Anti-DPPX antibody-associated encephalitis with the first detected anti-Yo antibody during one-year follow-up: a case report
Source: Front Immunol. 2026 Sep 18;17:1792230. doi: 10.3389/fimmu.2026.1792230 (PMC13630750; doi:10.3389/fimmu.2026.1792230)
Supplement: Supplementary file 2 [file Image2.pdf]

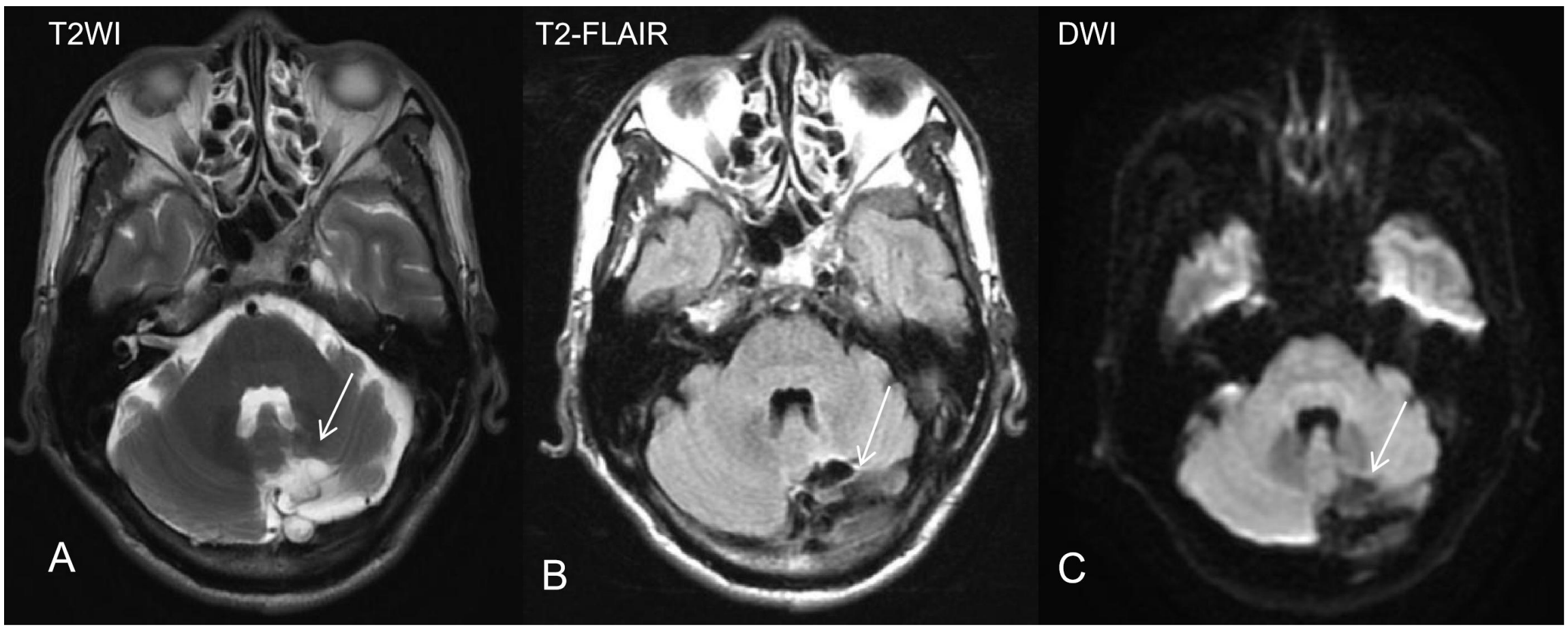


Supplementary Figure 2. MRI showed no acute infarction and confirmed left cerebellar ncephalomalacia near the vermis. A T2-weighted sequence shows hyperintensity in the left cerebellar hemisphere. B T2/FLAIR sequence reveals hypointensity in the left cerebellar hemisphere. C DWI sequence demonstrates hypointensity in the left cerebellar hemisphere.
